# Relationship between brain activity, cognitive function, and sleep spiking activation in new-onset self-limited epilepsy with centrotemporal spikes

**DOI:** 10.3389/fneur.2022.956838

**Published:** 2022-11-09

**Authors:** Yanzhang Li, Yihan Li, Jintao Sun, Kai Niu, Pengfei Wang, Yue Xu, Yingfan Wang, Qiqi Chen, Ke Zhang, Xiaoshan Wang

**Affiliations:** ^1^Department of Neurology, Nanjing Brain Hospital, Nanjing Medical University, Nanjing, China; ^2^MEG Center, Nanjing Brain Hospital, Nanjing, China

**Keywords:** electrical status epilepticus of sleep (ESES), magnetoencephalography (MEG), cognitive function, brain activity, self-limited epilepsy with centrotemporal spikes, sleep spiking activation

## Abstract

**Objective:**

This study aimed to investigate the relationship between cognitive function sleep spiking activation and brain activity in self-limited epilepsy with centrotemporal spikes (SeLECTS).

**Methods:**

We used spike-wave index (SWI), which means the percentage of the spike and slow wave duration to the total non-REM (NREM) sleep time, as the grouping standard. A total of 14 children with SeLECTS (SWI ≥ 50%), 21 children with SeLECTS (SWI < 50%), and 20 healthy control children were recruited for this study. Cognitive function was evaluated using the Wechsler Intelligence Scale for Children, Fourth Edition (Chinese version) (WISC-IV). Magnetic source activity was assessed using magnetoencephalography calculated for each frequency band using the accumulated source imaging (ASI) technique.

**Results:**

Children with SeLECTS (SWI ≥ 50%) had the lowest cognitive function scores, followed by those with SeLECTS (SWI < 50%) and then healthy controls. There were significant differences in the localization of magnetic source activity between the three groups: in the alpha (8–12 Hz) frequency band, children with SeLECTS (SWI ≥ 50%) showed deactivation of the medial frontal cortex (MFC) region; in the beta (12–30 Hz) frequency band, children with SeLECTS (SWI ≥ 50%) showed deactivation of the posterior cingulate cortex (PCC) segment; and in the gamma (30–80 Hz) frequency band, children in the healthy group showed activation of the PCC region.

**Conclusion:**

This study revealed significant decreases in cognitive function in children with SeLECTS (SWI ≥ 50%) compared to children with SeLECTS (SWI < 50%) and healthy children, as well as significant differences in magnetic source activity between the three groups. The findings suggest that deactivation of magnetic source activity in the PCC and MFC regions is the main cause of cognitive function decline in SeLECTS patients with some frequency dependence.

## Introduction

Self-limited epilepsy with centrotemporal spikes (SeLECTS), which is also known as Rolandic epilepsy (RE) and Benign childhood epilepsy with centrotemporal spikes (BECTS), is the most common type of childhood idiopathic epilepsy (1). The International League Against Epilepsy (ILAE) has changed the term “benign” to “self-limited” in the latest version of the definition in 2022, taking into account that the typical evolution of this type of epilepsy is age-related onset and remission (2, 3). In China, the mean age of onset of SeLECTS is 6.85 years and the male to female ratio is 6:4 (4). Typical clinical presentation is limited motor-sensory seizures on one side of the mouth and face, with extension to generalized tonic-clonic seizures (5). In these patients, electroencephalogram (EEG) commonly shows normal background activity and spike discharges in the centrotemporal region with high amplitude that are usually more pronounced during sleep (6). While SeLECTS was previously thought to have a favorable prognosis, a growing number of studies suggest that, even in adulthood when patients are seizure-free, SeLECTS is associated with greater neuropsychiatric dysfunction (7–9).

Children with SeLECTS show significantly more discharges during sleep, some of which reach electrical status epilepticus (ESES) (10). This group of patients is characterized by a poor prognosis and a negative impact on the development of cognitive function (11). It is widely believed that frequent epileptiform discharges affect the formation and establishment of synapses, damage neural circuits, and produce changes in the structure and function of brain tissue that affect the entire functional network of the brain and lead to cognitive decline (12–14). In a clinical study, Overvliet et al. (15) found an association between cognitive impairment and nocturnal epileptiform discharges. This association was interpreted as a disruption of the functional connections responsible for the corresponding cognitive ability. In a functional magnetic resonance imaging (fMRI) study of nine children with SeLECTS with ESES, we observed alterations in the salience network (SE) and central executive network (CEN) (10).

ESES is an interictal EEG pattern that occurs during slow wave sleep (16). ESES patterns usually consist of continuous, symmetrical spike patterns with variable discharge frequencies, usually in the range of 1.5–3 HZ. The spike-wave index (SWI), which is the percentage of the spike and slow wave duration to the total non-REM (NREM) sleep time, is commonly used to measure the severity of ESES (17). In general, the higher the SWI, the more epileptiform discharges and the more severe the cognitive impairment (18). It is now generally accepted that a SWI of ≥85% can be considered ESES, but some studies have also shown that cognitive and behavioral impairment can occur even with a low percentage of sleep. So some authors also use lower SWI values as a cut-off (19, 20), or consider that a SWI of at least ≥50 should trigger the possibility of an ESES related syndrome (21). In the current study, we decided to divide patients in two groups according to a SWI threshold at 50%, and all EEG interpretations were performed by two experienced EEG physicians.

Magnetoencephalography (MEG) is a non-invasive neuroimaging method with ultra-high spatial and temporal resolution (22). Compared to EEG, the magnetic field is not attenuated as it passes through the skull and scalp, allowing for clearer detection of brain magnetic activity (23). MEG can capture higher frequency information compared to fMRI, which can only retain low frequency information (24, 25). Given the frequency-dependent nature of brain activity (26), MEG can be used to more accurately localize neuromagnetic brain activity in each frequency band.

Previous studies have investigated differences in cognitive function in children with SeLECTS in longitudinal comparisons, as well as differences before and after medication administration (27–29). However, few studies have compared the cognitive function of children with SeLECTS without antiepileptic drugs (AEDs) in a cross-sectional manner with different evolutionary patterns, especially using the MEG technique (30, 31). We use EEG as a basis for grouping because children with developmental epileptic encephalopathy with spike-and-wave activation in sleep (DEE-SWAS) and epileptic encephalopathy with spike-and-wave activation in sleep (EE-SWAS), as defined by the latest ILAE definitions, whose diagnostic criteria include significant behavioral regression and negative myoclonus in addition to EEG manifestations, are difficult to diagnose at first presentation. Most of the patients diagnosed were already taking AEDs, which interfered with the results of this experiment. Therefore, in this trial we used only the EEG, an objective indicator, as a basis for grouping. In the present study, we compared the intensity and localization of magnetic sources in different frequency bands in children with SeLECTS (SWI ≥ 50%), children with SeLECTS (SWI < 50%), and healthy control (HC) children. Cognitive function in the three groups was assessed using the Wechsler Intelligence Scale for Children, Fourth Edition (Chinese version) (WISC-IV). We tentatively hypothesized that SeLECTS children (SWI ≥ 50%) would have the worst cognitive function and that there would be differences in magnetic source activity intensity and magnetic source localization between the three groups.

## Experimental procedures

### Participants

Between October 2020 and October 2021, 21 children were diagnosed with SeLECTS (SWI ≥ 50%) and 24 children were diagnosed with SeLECTS (SWI < 50%) only at the Neurology Department of the Nanjing Children's Hospital and Nanjing Brain Hospital. Of the SeLECTS (SWI < 50%) patients, two were taking AEDs and one had a history of encephalitis; thus, 21 children were included in this study. Of the SeLECTS patients (SWI ≥ 50%), six had a history of AEDs and one had an autism spectrum disorder; thus, only 14 patients were recruited for this study. We also recruited 20 HC children matched with the patients in terms of age, gender, parents educational background, and family socioeconomic status. The inclusion criteria for patients were as follows: (1) a diagnosis of SeLECTS according to the ILAE 2022 classification of epilepsy syndrome (32); (2) no use of AEDs at the time of enrollment; (3) normal brain MRI image; and (4) no other types of epilepsy or other major neuropsychiatric disorders. The exclusion criteria were: (1) did not cooperate when MEG and MRI data were collected which interfered with data collection; (2) brain MRI showed abnormalities; (3) a diagnosis of other types of epilepsy or major neuropsychiatric disorders; and (4) the patients parents did not cooperate with the examination or provide informed consent. All patients were seizure-free for at least 72 h before and 24 h after the MEG and MRI scans were performed.

Based on previous studies (19–21), we hypothesize that there is a significant difference between the cognitive function, brain activity of children with SeLECTS using SWI = 50% as the cut-off. In the SeLECTS (SWI ≥ 50%) group, two EEG results for patients within 1 month showing SWI ≥ 50%; the SWI (%) was obtained as the total number of minutes of all spike and slow-wave abnormalities divided by the total number of minutes of NREM and multiplied by 100 (33). All patients were subjected to sleep deprivation to better record the EEG during sleep. The recording time was 2 h and the SWI was calculated on 45 min of NREM sleep. EEG reports for all patients issued by two experienced EEG physicians.

This study was approved by the Medical Ethics Committee of Nanjing Medical University, Nanjing Brain Hospital and Nanjing Children's Hospital. In addition, the parents of all participants in the study provided informed consent.

### Neuropsychological assessment

For all participants, cognitive function was assessed using the Wechsler Children Intelligence Scale, Fourth Edition (Chinese version) (WISC-IV). The test comprises 14 sub-tests that provide a full-scale intelligence quotient (FSIQ) to account for overall cognitive abilities as well as four additional composite scores that account for cognitive abilities in different areas (34). The four composite scores are the Verbal Comprehension Index (VCI), Perceptual Reasoning Index (PRI), Working Memory Index (WMI), and Processing Speed Index (PSI). The VCI, which includes tests of analogy, vocabulary, comprehension, and general knowledge, measures language learning ability, concept formation, abstract thinking, and analytical generalization. The PRI includes block design, picture concepts, matrix reasoning, and fill-in-the-blank tests, which measure reasoning ability, spatial perception, and visual organization, respectively. The WMI includes a recited number test, alphabetic-numeric alignment, and arithmetic test to measure memory ability, comprehension, and application of external information. The PSI includes decoding tests, symbol retrieval, and scratch tests to measure the speed of understanding simple information and the speed and accuracy of recording (35, 36).

### MEG recordings

MEG data were collected in the magnetic shielding room of the MEG Center of Nanjing Brain Hospital using a full-head CTF-275 channel MEG system (VSM Medical Technology Company, Canada). In preparation for data collection, subjects were asked to make sure they were free of any metal objects and to fix three coils at the base of their nose and in front of each ear to determine the position of their head in the MEG system. During data collection, subjects were asked to remain relaxed, stay still, keep their eyes closed, stay awake, and keep their mouth slightly open. If a subject's head moved more than 5 mm during the data collection process, the data were discarded and re-collected. The sampling frequency of MEG was 6,000 Hz and at least six 120 s sessions of MEG data were collected per subject. Noise was eliminated using third-order gradients before data acquisition.

### MRI scan

All participants underwent a 3.0 T MRI (Siemens, Germany) scan after MEG data collection. Before MRI scanning, coils similar to those used in MEG data collection were placed at the nose root and in front of both ears to locate each subjects head position.

### Data preprocessing

MEG data were processed through the following steps: First, we excluded MEG waveforms with significant noise and artifacts (amplitude > 6 pT), based on previous studies (37). Next, we filtered the MEG data in the frequency range 1–70 Hz and used it to identify high amplitude peaks in SeLECTS. To reduce the impact of spikes, we selected MEG data without spikes for a continuous period of 60 s for processing. Finally, we analyzed the six frequency bands of the selected waveform, namely delta (1–4 Hz), theta (4–8 Hz), alpha (8–12 Hz), beta (12–30 Hz), gamma (30–80 Hz), and ripple (80–250 Hz). The data were filtered before analysis to avoid environmental alternating current (AC) power interference around the 50 Hz band. MEG data from all subjects were analyzed using the MEG processor (https://sites.google.com/site/braincloudx/).

### Source localization

Based on previous studies (38–40), we used accumulated source imaging (ASI) to analyze neuromagnetic source activity in multiple frequency bands in the selected MEG data. The sum of the volumes of source activity over a period of time is defined as ASI, which can locate the neuromagnetic sources. ASI can be expressed by the following equation:


$$\begin{array}{l} \text{Asi}(r,s)=\sum_{t=1}^{t=n}Q(r,t) \end{array}$$

Where, ASI represents the accumulated source strength at position r, s indicates the time slice, n represents the time point of the MEG data, and Q represents the source activity at source r and at time point t. We defined s ≥ 1 and s ≤ n/2.

We used two-step beamforming to calculate the source activity and locate the source (37). First, we calculated the lead domain for each source (or voxel), which is called a voxel-based partial sensor, and generated the MEG data matrix. Next, to minimize the influence of coherent sources in the localization of the source, we performed a partial sensor overlay for each voxel (37). We then calculated the covariance of all sensors. We calculated two sets of source images using the vector beamformer and estimated the coherent source and source direction using the variance matrix vector beamformer (41). After these steps, we used the scalar beamformer to generate the source activity (or virtual sensor waveform). The algorithm has been described in detail and validated in prior studies (41).

For each subject, the entire brain was scanned at a resolution of 6 mm. In cases where the distance between two voxels was < 10 mm, they were considered as one source. The individual MRI data were merged with the MEG data by fixing the nasal root and three points in front of the ear at the time of data acquisition. This approach allows us to segment and visualize brain regions.

### Statistical analysis

Fisher's exact probability method was used to examine the localization of the main neuromagnetic sources in the three groups. One-way analysis of variance (ANOVA) was performed on the source strength, Wechsler scale scores, and subject age. We also conducted a homogeneity test of variance. We compared the age at onset and duration of disease between the SeLECTS (SWI ≥ 50%) group and SeLECTS (SWI < 50%) group using the two-sample *t-*test. Pearson or Spearman correlation analysis was used to analyze the relationship between the intensity of the magnetic source and the clinical characteristics of each group of frequency bands. The level of statistical significance was set at *p* < 0.05 with Bonferroni multiple comparison correction (e.g., for six bands in three groups, *p* < 0.002). All statistical analyses were performed using SPSS version 24.0 for Windows (SPSS Inc., Chicago, IL, USA).

## Results

### Participants

The final sample included 14 children with SeLECTS (SWI ≥ 50%) including eight females, with a mean age of 96.94 ± 13.68 months, a mean age of onset of 90.89 ± 15.08 months, a mean disease duration of 6.05 ± 6.06 months, and an average of 2.64 ± 1.45 seizures. The 21 children with SeLECTS (SWI < 50%), which included 10 males, had a mean age of 102.59 ± 19.20 months, a mean age of onset of 97.84 ± 16.25 months, a mean disease duration of 5.22 ± 6.68 months, and an average of 2.00 ± 1.18 seizures. The statistical analysis showed no significant differences between the two groups in terms of gender, age, age of onset, disease duration, or number of seizures. The detailed data of the participants are shown in Table 1.

**Table 1:** Demographic and clinical characteristics of the included participants.

**Participant**	**Gender**	**Age (months)**	**Epilepsy duration (months)**	**Age at onset (months)**	**Number of seizures**	**SWI**
1^a^	F	106	5.8	100.2	4	50–60%
2^a^	M	86.1	5.8	80.3	2	55%
3^a^	F	105.5	2	103.5	2	65%
4^a^	M	103.2	4.4	98.8	3	60%
5^a^	F	97.2	24	73.2	5	65–70%
6^a^	M	72.6	8.3	64.3	1	70%
7^a^	F	82.4	2.5	79.9	6	70%
8^a^	F	112.5	12.5	100	2	50%
9^a^	F	116.3	2.6	113.7	2	50%
10^a^	M	95.9	3	92.9	3	60%
11^a^	F	117	2.5	114.5	2	65%
12^a^	F	91.8	2.2	89.6	1	50–60%
13^a^	M	85.4	8	77.4	2	60%
14^a^	M	85.2	1.1	84.1	2	50%
15	F	84.8	12	82.8	2	40%
16	M	120.6	12	108.6	3	30%
17	F	132.8	24	108.8	2	30%
18	M	114.5	5.4	109.1	5	35%
19	F	73.5	4	69.5	4	30%
20	M	101.5	1	100.5	2	30%
21	F	99.5	4	95.5	2	25%
22	M	107.3	9.3	98	1	40%
23	M	85	1.8	83.2	1	20%
24	F	99.4	0.6	98.8	1	20%
25	F	74.6	0.7	73.9	1	40%
26	M	131.8	0.6	131.2	3	30%
27	F	96.9	0.6	96.3	1	10%
28	M	135.8	18.4	117.4	1	10%
29	M	110.8	10	100.8	2	15%
30	M	76.8	2.8	74	4	25%
31	F	113.3	0.4	112.9	2	40%
32	M	77.5	0.3	77.2	2	40%
33	F	99.4	0.6	98.8	1	20%
34	F	101.1	0.3	100.8	1	40%
35	F	117.4	0.9	116.5	1	35%

SWI, spike-wave index; ^*a*^SeLECTS (SWI ≥ 50%) group.

### WISC-IV scores

**Figure 1:**
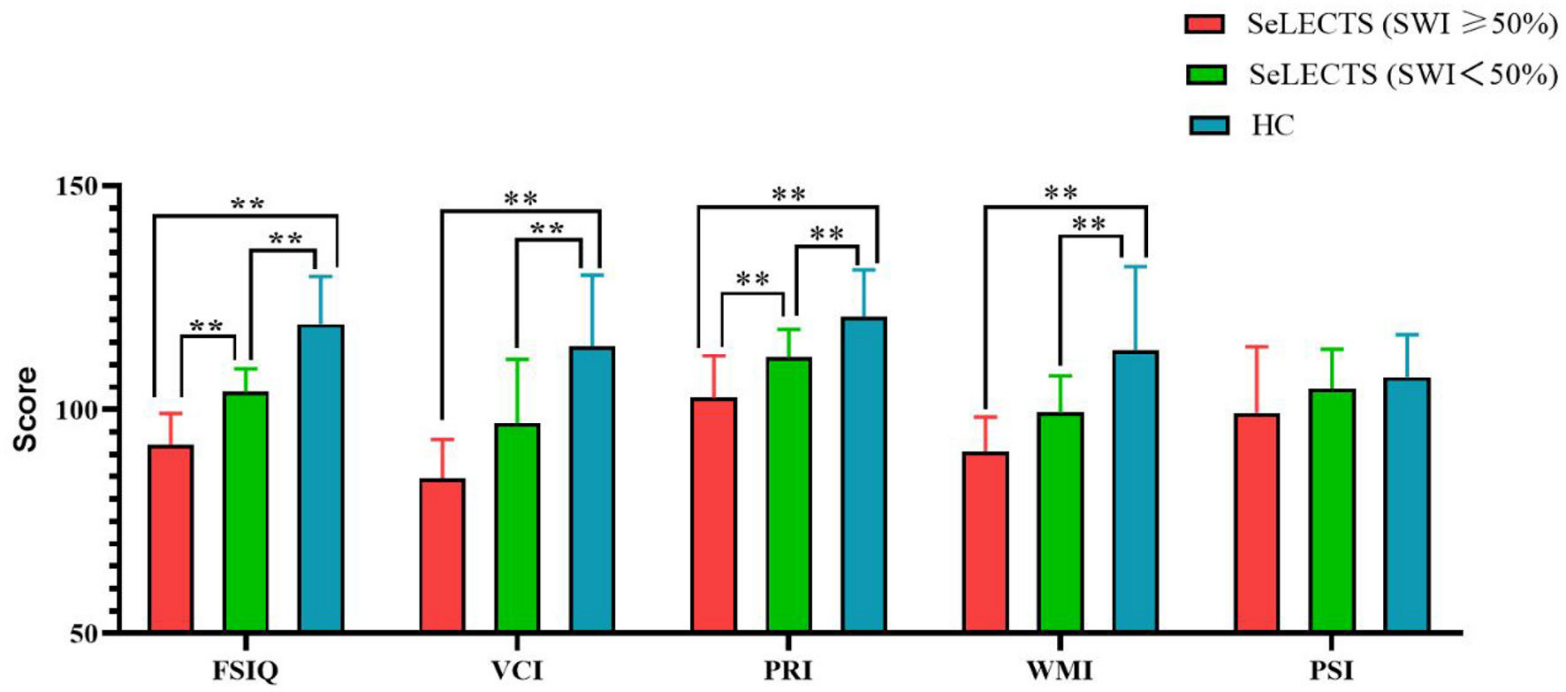
Comparison of WISC-IV scores between the SeLECTS (SWI ≥ 50%), SeLECTS (SWI < 50%), and HC groups. FSIQ, Full-scale Intelligence Quotient; VCI, verbal comprehension index; PRI, perceptual reasoning index; WMI, working memory index; PSI, processing speed index. **, The *p*-value was statistically significant.

As shown in Table 2, there were statistically significant differences between the three groups in terms of total score and PRI, with the SeLECTS (SWI ≥ 50%) group having the lowest score followed by the SeLECTS (SWI < 50%) group; the HC group had the highest score. In terms of VCI and WMI, both the SeLECTS (SWI ≥ 50%) group and SeLECTS (SWI < 50%) group had significantly lower scores than the HC group, but there was no statistically significant difference between the scores of the SeLECTS (SWI ≥ 50%) group and SeLECTS (SWI < 50%) group. There were no significant group differences between the PSI scores. Additional details of the performance for each group are provided in Figure 1.

**Table 2:** Comparison of WISC-IV scores between the SeLECTS (SWI ≥ 50%), SeLECTS (SWI < 50%), and HC groups.

**WISC-IV**	**SeLECTS (SWI ≥50%) (Group 1, *n* = 14)**	**SeLECTS (SWI < 50%) (Group 2, *n* = 21)**	**HC** **(Group 3, *n* = 20)**	***p***	***P*: Group 1** **vs. Group 2**	***P*: Group 1** **vs. Group 3**	***P*: Group 2** **vs. Group 3**
FSIQ	92.21 ± 6.879	103.94 ± 5.117	119.10 ± 10.586	0.00*	0.000**	0.000**	0.000**
VCI	84.64 ± 8.705	96.94 ± 14.263	114.1 ± 15.864	0.00*	0.019	0.000**	0.004**
PRI	102.57 ± 9.387	111.65 ± 6.214	120.7 ± 10.489	0.00*	0.007**	0.000**	0.004**
WMI	90.64 ± 7.652	99.29 ± 8.252	113.3 ± 18.576	0.00*	0.076	0.000**	0.002**
PSI	99.21 ± 14.818	104.71 ± 8.73	107.15 ± 9.549	0.125	0.553	0.253	0.807

FSIQ, Full-scale Intelligence Quotient; VCI, verbal comprehension index; PRI, perceptual reasoning index; WMI, working memory index; PSI, processing speed index.

* p < 0.05 after one-way analysis of variance (ANOVA);

** p < 0.0167 after Bonferroni correction for multiple comparisons.

### Source location

**Figure 2:**
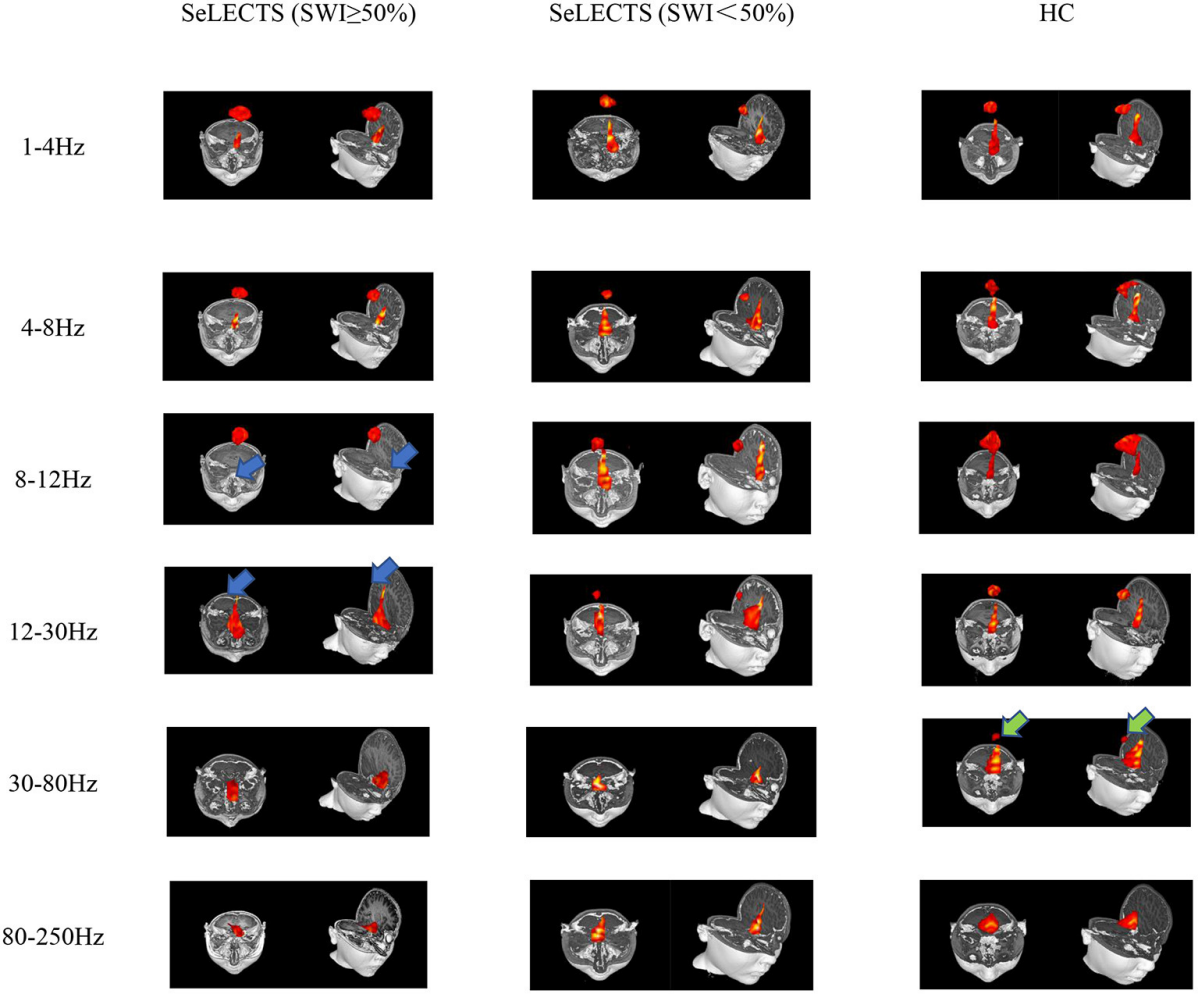
Typical magnetic source localization in the frequency band of 1–250 Hz for three groups of subjects. In the 8–12 and 12–30 Hz frequency bands, the SeLECTS (SWI ≥ 50%) group was inactivated in the PCC region, as indicated by the blue arrows. In the 30–80 Hz band, the HC group is activated in the PCC region as shown by the green arrow.

According to the whole brain accumulative magnetic source imaging, there were 1–3 primary magnetic source locations per subject in the resting state (Figure 2), including the peri-Rolandic area (PR), posterior cingulate cortex (PCC), medial frontal cortex (MFC), medial temporal lobe (MTL), and deep brain area (DBA). In the low frequency band (<80 Hz), there was a significant difference in source localization between the three groups; however, in the high frequency band (>80 Hz), there was not. Specifically, in the alpha (8–12 Hz) band, the SeLECTS (SWI < 50%) group and HC group were mainly localized in the MFC and PCC regions, while the magnetic source in the SeLECTS (SWI ≥ 50%) group was mainly localized in the PCC region. In the gamma band (30–80 Hz), the SeLECTS (SWI ≥ 50%) group and SeLECTS (SWI < 50%) group were mainly localized in the MFC region, while the HC group was mainly localized in the MFC and PCC regions. In the beta (12–30 Hz) frequency band, we found group differences in localization in the PCC region. *Post-hoc* analysis showed a statistically significant difference in localization in the SeLECTS (SWI ≥ 50%) group compared to the SeLECTS (SWI < 50%) group and HC group, but no statistically significant difference between the SeLECTS group and healthy controls.

We did not find significant differences in the other three frequency bands. However, as shown in Figure 2, in the higher frequency bands the magnetic source activity is more frequent in the DBA region than in the lower frequency bands, while in the lower frequency bands the magnetic source activity in the PR region is more frequent than in the higher frequency bands. The localization and statistical comparison of the main magnetic sources in each frequency band for the three groups are detailed in Table 3.

**Table 3:** Comparison of predominant neuromagnetic activity in the SeLECTS (SWI ≥ 50%), SeLECTS (SWI < 50%), and HC groups.

**Frequency band (Hz)**	**Source location**	**SeLECTS** **(SWI ≥50%)** **(Group 1, *n* = 14)**	**SeLECTS (SWI < 50%) (Group 2, *n* = 21)**	**HC** **(Group 3, *n* = 20)**	***p***	***P*: Group 1 vs. Group 2**	***P*: Group 1 vs. Group 3**	***P*: Group 2 vs. Group 3**
1–4 Hz	PR	3	5	2	0.556			
	PCC	5	5	10	0.222			
	MFC	6	16	13	0.143			
	MTL	2	1	2	0.623			
	DBA	1	2	2	1.000			
4–8 Hz	PR	3	4	2	0.723			
	PCC	4	11	9	0.404			
	MFC	6	13	13	0.451			
	MTL	3	1	0	0.078			
	DBA	2	2	1	0.839			
8–12 Hz	PR	2	3	4	0.902			
	PCC	9	14	12	0.937			
	MFC	3	15	15	0.003*	0.006**	0.004**	1.000
	MTL	2	0	0	0.061			
	DBA	2	2	1	0.839			
12–30 Hz	PR	4	3	3	0.608			
	PCC	1	11	11	0.009*	0.01**	0.009**	1.000
	MFC	6	16	14	0.118			
	MTL	2	2	0	0.287			
	DBA	1	4	2	0.675			
30–80 Hz	PR	0	0	0	-			
	PCC	1	1	10	0.001*	1.000	0.011**	0.001**
	MFC	11	19	18	0.596			
	MTL	1	3	0	0.231			
	DBA	2	6	3	0.522			
80–250 Hz	PR	0	0	0	-			
	PCC	0	0	0	-			
	MFC	10	11	11	0.541			
	MTL	3	8	6	0.617			
	DBA	4	7	5	0.929			

PR, peri-Rolandic area; PCC, posterior cingulate cortex; MFC, medial frontal cortex; MTL, medial temporal lobe; DBA, deep brain area.

* p < 0.05 after Fishers exact test;

** p < 0.0167 after Bonferroni correction for multiple comparisons. The number in the columns represents the number of patients showing this source localization.

### Source strength

In the delta (1–4 Hz) frequency band, the magnetic source intensity of the SeLECTS (SWI ≥ 50%) group was significantly higher than that of the SeLECTS (SWI < 50%) group and HC group. However, there was no significant difference between the magnetic source intensity of the SeLECTS (SWI < 50%) group and HC group. In the alpha (8–12 Hz) and gamma (30–80 Hz) frequency bands, we found a statistically significant difference in the magnetic source intensity between the three groups; however, the pairwise comparison showed no statistically significant difference between the two groups. In the other three frequency bands, there were no statistically significant group differences. Detailed statistical analysis of the neuromagnetic sources is shown in Table 4.

**Table 4:** Comparison of neuromagnetic source strength between the SeLECTS (SWI ≥ 50%), SeLECTS (SWI < 50%), and HC groups.

**Frequency band (Hz)**	**SeLECTS (SWI ≥50%)** **(Group 1, *n* = 14)**	**SeLECTS (SWI < 50%)** **(Group 2, *n* = 21)**	**HC** **(Group 3, *n* = 20)**	***p***	***P*: Group 1** **vs. Group 2**	***P*: Group 1** **vs. Group 3**	***P*: Group 2** **vs. Group 3**
1–4 Hz	89.97 ± 9.13	78.52 ± 5.64	76.34 ± 4.29	0.00*	0.001**	0.000**	0.432
4–8 Hz	79.32 ± 6.18	83.8 ± 5.3	81.55 ± 5.48	0.385	0.461	0.641	0.913
8–12 Hz	86.31 ± 12.38	79.10 ± 6.87	89.91 ± 5.61	0.05*	0.355	0.191	0.015
12–30 Hz	72.64 ± 5.28	72.82 ± 7.52	70.51 ± 4.28	0.414	0.931	0.311	0.221
30–80 Hz	53.86 ± 3.7	51.72 ± 4.54	56.51 ± 7.21	0.03*	0.357	0.429	0.048
80–250 Hz	39.21 ± 2.04	39.79 ± 3.2	41.29 ± 4.35	0.187	0.888	0.201	0.521

* p < 0.05 after one-way analysis of variance (ANOVA);

** p < 0.002 after Bonferroni correction for multiple comparisons.

### Clinical correlation

Our analysis revealed no significant correlation between the magnetic source intensity and clinical data in the SeLECTS (SWI ≥ 50%) group and SeLECTS (SWI < 50%) group. Similarly, there was no significant correlation between the age of onset and disease duration in the two groups.

## Discussion

This study has revealed significant differences in neuropsychological evaluation results between unmedicated children with SeLECTS (SWI < 50%) and unmedicated children with SeLECTS (SWI ≥ 50%). Similarly, there were significant differences in magnetic source localization and magnetic source intensity between these two groups compared to the HC group, and they showed some frequency dependence. This is tentatively consistent with our hypothesis that frequent discharges during sleep would alter normal brain magnetic source activity and thus affect cognitive function. To the best of our knowledge, this is the first MEG study of unmedicated SeLECTS (SWI < 50%), unmedicated SeLECTS (SWI ≥ 50%), and HC subjects, as previous studies have mainly used fMRI and focused on relevant functional networks of the brain (42–44).

### Clinical data and characteristics

Previous studies (10, 44) suggest that children with SeLECTS (SWI ≥ 50%) have an earlier age of onset, longer disease duration, and more seizures than children with SeLECTS (SWI < 50%). However, we found no such patterns in our sample. During patient recruitment, we found that parents who become more aware of epilepsy disorders are inclined to go to the hospital immediately for even minor petit mal seizures or nocturnal episodes. This means that, regardless of the outcome of SeLECTS, in most cases patients were recruited at the time of the first one or two seizures, resulting in no statistically significant difference between the two groups in terms of duration of illness and number of seizures. However, at follow-up, we found that children with SeLECTS (SWI ≥ 50%) were not as well controlled as children with SeLECTS (SWI < 50%), even with use of AEDs, and continued to have seizures. This further confirms that children with SeLECTS (SWI ≥ 50%) have poorer outcomes, more difficult seizure control, and lower neuropsychological scores (42).

We found no significant difference in the age of onset of SeLECTS (SWI ≥ 50%) compared to SeLECTS (SWI < 50%) children, which is likely because of the sample size. In addition, we did not find a significant correlation between the age at onset and duration of illness. We do not believe that there is a simple linear relationship between magnetic source intensity, age at onset, number of episodes, and duration of illness, which we plan to reconfirm in a larger sample (45).

### Neuropsychological test results

We found that SeLECTS (SWI < 50%) patients and SeLECTS (SWI ≥ 50%) scored significantly lower than healthy controls on both the VCI and WMI, although there was no significant difference between the two patient groups. This finding is similar to the results of previous scales assessing SeLECTS patients with unilateral and bilateral discharges (46). In the SeLECTS (SWI ≥ 50%) group, four patients had bilateral discharges; in the SeLECTS (SWI < 50%) group, all 21 had unilateral discharges. However, we did not find a correlation between SeLECTS (SWI ≥ 50%) and bilateral discharges. We hope to expand the sample size in a future study. In terms of total score and PRI, not only did the SeLECTS (SWI ≥ 50%) and SeLECTS (SWI < 50%) group score significantly lower than the HC group, the SeLECTS (SWI ≥ 50%) group also scored significantly lower than the SeLECTS (SWI < 50%) group. There is growing evidence (44, 47, 48) that, because the brain is at a critical stage of development during childhood, frequent epileptiform discharges during this period can affect the formation and development of neural protrusions, leading to widespread cognitive impairment in children with SeLECTS. The neural network theory of epilepsy suggests that abnormal discharges during the interictal period may affect the neuropsychological development of children more than the clinical seizures themselves (9, 49, 50). This could explain why children with SeLECTS (SWI ≥ 50%) have worse cognitive function than SeLECTS (SWI < 50%) patients and healthy controls (42, 44); the higher the number of discharges during the interictal period, the greater the impact on cognitive function.

Our findings showed no significant difference in PSI between the SeLECTS (SWI ≥ 50%) group, SeLECTS (SWI < 50%) group, and healthy controls, which is inconsistent with previous studies of cognitive function in patients with initially diagnosed SeLECTS (35). However, some studies have reported non-significant differences between SeLECTS (SWI < 50%) patients and healthy controls in terms of PSI (46). A follow-up study of SeLECTS patients taking medication for 1 year found no significant increase in PSI over the1 year period (51). In terms of overall trends, we found that the PSI scores and mean scores of children with SeLECTS (SWI ≥ 50%) and SeLECTS (SWI < 50%) were lower than those of healthy controls, but it did not reach the level of statistical significance. Thus, we plan to expand the sample size for further research.

### Source localization

The magnetic source analysis revealed that the SeLECTS (SWI ≥ 50%) group showed extensive deactivation of the PCC and MFC regions, specifically in the MFC region in the alpha (8–12 Hz) frequency band and in the PCC region in the beta (12–30 Hz) frequency band. In addition, in the gamma (30–80 Hz) frequency band, the SeLECTS (SWI ≥ 50%) group and SeLECTS (SWI < 50%) group showed inactivation in the PCC region. Although a clear mechanism of cognitive decline in children with SeLECTS has not been identified, some studies suggest that frequent interictal discharges can lead to altered magnetic sources in important brain regions, thus affecting the corresponding functions (40, 52, 53).

The brain functions with network properties that dynamically regulate information interactions between various systems. The default mode network (DMN) is an important network that maintains the basic state of the nervous system, remaining active during resting states and being inhibited during working states or in the presence of external stimuli. The DMN consists mainly of discrete, bilateral, and symmetrical cortical areas located in the medial and lateral parietal, medial prefrontal, and medial and lateral temporal cortices of the brain (54, 55). The MFC and PCC are well-known as core regions of the DMN, and the SeLECTS (SWI ≥ 50%) and SeLECTS (SWI < 50%) groups showed deactivation of MFC and PCC regions in the corresponding frequency bands, suggesting that DMN impairment is an important cause of cognitive decline in children with SeLECTS (56, 57).

Previous studies have found that prefrontal regions in patients with SeLECTS are associated with neuropsychological deficits (10). A study of brain white matter in patients with SeLECTS similarly found that reduced function of frontal regions may be responsible for cognitive impairment (58). In the present study, the SeLECTS (SWI ≥ 50%) group under the alpha frequency band showed deactivation in the MFC region compared to the SeLECTS (SWI < 50%) and HC groups, which explains why the SeLECTS (SWI ≥ 50%) group had poorer cognitive function. Similarly, a study by Li et al. (45) found that magnetic source activity in children with SeLECTS with poorer cognitive function was deactivated in the MFC region.

In a study of changes in brain magnetic activity and cognitive function in SeLECTS patients before and after AEDs, it was found that children with SeLECTS showed activation of the PCC region in several frequency bands after treatment, especially the gamma frequency band. Treated SeLECTS patients also showed significant improvement in VCI and PRI scores, suggesting that PCC regions are closely related to verbal comprehension and perceptual reasoning functions (51). Similarly, our study found that both the SeLECTS (SWI ≥ 50%) and SeLECTS (SWI < 50%) groups were inactivated in the PCC region in the gamma frequency band compared to the HC group. In the beta frequency band, only the SeLECTS (SWI ≥ 50%) group showed deactivation in the PCC region, while the SeLECTS (SWI < 50%) and HC groups showed activation, suggesting that PCC deactivation in multiple frequency bands affects, not only a specific band, affects patients cognitive function.

Previous studies (55, 56) suggest that network reorganization of the DMN leads to altered cognitive function in SeLECTS patients. Thus, in the present study, we hypothesized a causal relationship between magnet source inactivation and network reorganization, and that magnet source inactivation would lead to impaired corresponding functional connectivity of the DMN (45). In addition, we found no significant difference in the number of seizures between the SeLECTS (SWI ≥ 50%) and SeLECTS (SWI < 50%) groups. However, there was a significant difference in cognitive function between the two groups, which suggests that frequent nighttime sleep discharges are the main cause of cognitive decline in SeLECTS patients, rather than seizures.

Notably, not only in the SeLECTS group, but also in a MEG study of Childhood absence epilepsy (CAE), we found that the higher the frequency band, the more the magnetic source activity in epileptic patients tended to be distributed in the deep brain; the exact mechanism of this has not yet been clarified (40). In the future, we plan to analyze magnetic source activity in higher frequency bands by expanding the sample size and using more sophisticated MEG processing software.

### Source strength

In the delta (1–4 Hz) frequency band, the magnetic source intensity in the SeLECTS (SWI ≥ 50%) group was significantly higher than that in the SeLECTS (SWI < 50%) group and HC group. In a prior MEG study (35) examining the cognitive function of children with SeLECTS not taking AEDs, we found that, in the gamma (30–80 Hz) frequency band, the magnetic source activity intensity was highest in SeLECTS children in the group with poorer cognitive function. These results are consistent with a previous MEG study (41) showing that increased magnetic source intensity in children with epilepsy during the interictal period was attributed to frequent discharges. Another MEG study (53) of interictal discharges in SeLECTS confirmed our finding that patients with epilepsy who had interictal discharges had higher magnetic source intensity in the delta (1–4 Hz) frequency band.

### Limitations

This study is subject to several limitations. Firstly, the sample size for each group was relatively small. We plan to expand the sample size to validate the experimental results in future studies. Secondly, because unable to assess the patient's eventual cognitive decline, we were making impossible to make a diagnosis of DEE-SWAS and EE-SWAS, and we will more fully document the patient's seizures and subsequent cognitive decline in a follow-up study. As a third point, we artifacts in EMG, ECG, and other signals may interfere with the acquisition of MEG information, although we utilized various methods to eliminate these effects. Finally, the technology of the magnetic source imaging software used in this study is limited; therefore, we need to use other magnetic source imaging software in follow-up research to validate these results.

## Conclusion

This study investigated the relationship between magnetic brain activity and cognitive function in children with SeLECTS (SWI ≥ 50%) compared to SeLECTS (SWI < 50%) patients and healthy controls. We found a significant decrease in cognitive function in the SeLECTS (SWI ≥ 50%) group compared to the SeLECTS (SWI < 50%) and HC groups, and a significant difference in magnetic source activity between the three groups. Deactivation of magnetic source activity in the PCC and MFC regions was the main cause of cognitive function decline in SeLECTS patients and showed some frequency dependence. We intend to follow up with these patients and to clarify the prognosis of SeLECTS (SWI ≥ 50%) vs. SeLECTS (SWI < 50%) and the corresponding changes in magnetic brain activity.

## Data availability statement

The raw data supporting the conclusions of this article will be made available by the authors, without undue reservation.

## Ethics statement

The studies involving human participants were reviewed and approved by the Medical Ethics Committee of Nanjing Medical University, Nanjing Brain Hospital and Nanjing Children's Hospital. Written informed consent to participate in this study was provided by the patients/participants' legal guardian/next of kin. Written informed consent was obtained from the minor(s)' legal guardian/next of kin for the publication of any potentially identifiable images or data included in this article.

## Author contributions

YaL, YiL, JS, and XW designed the research. YaL, YX, KN, and PW recruited the participants and analyzed the data. YaL, YW, KZ, and QC acquired the images. XW revised the manuscript. All authors approved the final submitted version and agreed to be accountable for its content.

## Funding

This study was supported by the General Program of Natural Science Foundation of Jiangsu Province (Grant No. BK20191127), the Health Department of Jiangsu Province (Grant No. H2018062), the Medical and Health International Cooperation Project of Nanjing Municipal Science and Technology Bureau (Grant No. 201911044), and the National Natural Science Foundation of China (Grant No. 82071455).

## Conflict of interest

The authors declare that the research was conducted in the absence of any commercial or financial relationships that could be construed as a potential conflict of interest.

## Publisher's note

All claims expressed in this article are solely those of the authors and do not necessarily represent those of their affiliated organizations, or those of the publisher, the editors and the reviewers. Any product that may be evaluated in this article, or claim that may be made by its manufacturer, is not guaranteed or endorsed by the publisher.
